# Dynamic involvement of ATG5 in cellular stress responses

**DOI:** 10.1038/cddis.2014.428

**Published:** 2014-10-23

**Authors:** H H Lin, S-M Lin, Y Chung, S Vonderfecht, J M Camden, P Flodby, Z Borok, K H Limesand, N Mizushima, D K Ann

**Affiliations:** 1 Department of Molecular Pharmacology, Beckman Research Institute, City of Hope, Duarte, CA, USA; 2 Division of Comparative Medicine, Beckman Research Institute, City of Hope, Duarte, CA, USA; 3 Department of Biochemistry, University of Missouri, Columbia, MO, USA; 4 Division of Pulmonary, Critical Care and Sleep Medicine, Department of Medicine, Will Rogers Institute Pulmonary Research Center, Keck School of Medicine, University of Southern California, Los Angeles, CA, USA; 5 Department of Biochemistry and Molecular Biology, Keck School of Medicine, University of Southern California, Los Angeles, CA, USA; 6 Department of Nutritional Sciences, The University of Arizona, Tucson, AZ, USA; 7 Department of Biochemistry and Molecular Biology, The University of Tokyo, Bunkyo-ku, Tokyo, Japan; 8 Irell & Manella Graduate School of Biological Sciences, Beckman Research Institute, City of Hope, Duarte, CA, USA

## Abstract

Autophagy maintains cell and tissue homeostasis through catabolic degradation. To better delineate the *in vivo* function for autophagy in adaptive responses to tissue injury, we examined the impact of compromised autophagy in mouse submandibular glands (SMGs) subjected to main excretory duct ligation. Blocking outflow from exocrine glands causes glandular atrophy by increased ductal pressure. *Atg5*^*f/−*^*;Aqp5-Cre* mice with salivary acinar-specific knockout (KO) of autophagy essential gene *Atg5* were generated. While duct ligation induced autophagy and the expression of inflammatory mediators, SMGs in *Atg5*^*f/−*^*;Aqp5-Cre* mice, before ligation, already expressed higher levels of proinflammatory cytokine and *Cdkn1a/p21* messages. Extended ligation period resulted in the caspase-3 activation and acinar cell death, which was delayed by *Atg5* knockout. Moreover, expression of a set of senescence-associated secretory phenotype (SASP) factors was elevated in the post-ligated glands. Dysregulation of cell-cycle inhibitor CDKN1A/p21 and activation of senescence-associated *β*-galactosidase were detected in the stressed SMG duct cells. These senescence markers peaked at day 3 after ligation and partially resolved by day 7 in post-ligated SMGs of wild-type (WT) mice, but not in KO mice. The role of autophagy-related 5 (ATG5)-dependent autophagy in regulating the tempo, duration and magnitude of cellular stress responses *in vivo* was corroborated by *in vitro* studies using MEFs lacking ATG5 or autophagy-related 7 (ATG7) and autophagy inhibitors. Collectively, our results highlight the role of ATG5 in the dynamic regulation of ligation-induced cellular senescence and apoptosis, and suggest the involvement of autophagy resolution in salivary repair.

Autophagy is a catabolic process that has an essential role in cellular adaptation to multiple types of stress by recycling of superfluous cellular material, safeguarding quality control in organelles, removing protein aggregates, and eliminating intracellular pathogens.^1^ Conceptually, autophagy serves a pro-survival mechanism by providing sources of energy and biosynthetic building blocks during starvation, removing dysfunctional organelles and large aggregates toxic to cells to avoid unwarranted cell death. However, upon sustained stress conditions, cell death eventually takes place either by excessive autophagy or by the induction of apoptosis and/or necrosis pathways.^2^ The ATG5, autophagy-related 5, has a pivotal role in autophagosome formation. Mouse neonates systemic deficient for ATG5 die within a day of birth,^3^ whereas mice depleted of *Atg5* in selected tissues have abnormalities ranging from neurodegeneration^4^ and age-related cardiomyopathy^5^ to liver tumors.^6^

Autophagy and senescence are two distinct, however functionally intertwined, cellular responses to stress.^7^ Cellular senescence is a state of stable growth arrest that is induced by telomere shortening, DNA-damage, oncogenes or other stresses. In general, senescence is a heterogeneous phenotype, which is characterized by a senescent-associated secretory phenotype (SASP), expression of senescence-associated *β*-galactosidase (SA-*β*-gal) and other senescent markers, and increased cell size.^8^ In culture system, inhibiting or enhancing autophagy leads to the opposite effect on premature senescence.^9, 10, 11, 12^ While premature senescence can be induced by a plethora of cell-extrinsic and cell-intrinsic stressors,^13^ little is known about the possible role of autophagy in modulating injury-induced cellular senescence *in vivo*. Rodent salivary duct ligation has been used as an experimental model system to study salivary gland atrophy, which often occurs in patients with Sjögren's syndrome or receiving head and neck radiation therapy. Although autophagy induction has been implicated in the repair of rapamycin-treated, post-ligated salivary glands,^14,15^ the roles played by autophagy in regulating the injury responses in submandibular glands (SMGs) have not been explored.

To explore how autophagy contributes to salivary (patho)physiology, we established a transgenic mouse model deficient for ATG5 in the salivary acinar cells. Previously, we have identified a role for basal autophagy in salivary homeostatic mechanisms that restrict acinar cell size and the number of secretory granules.^16^ Here, we report that ligation of the major SMG excretory duct triggers the glandular atrophy and the induction of autophagy. By comparing the acute and subacute stress responses from autophagy-impaired and -competent SMGs with duct obstruction, we established the intrinsic roles of ATG5-dependent autophagy in modulating salivary inflammatory responses, stress-induced senescence and cell death, which all occur sequentially in response to tissue injury. Our results provide *in vivo* evidence that stress-induced autophagic response is indispensable for resolving premature senescence in duct cells of the ligated glands, whereas ATG5 deficiency leads to delayed acinar cell death.

## Results

### Acinar-specific autophagy deficiency

**Figure 1:**
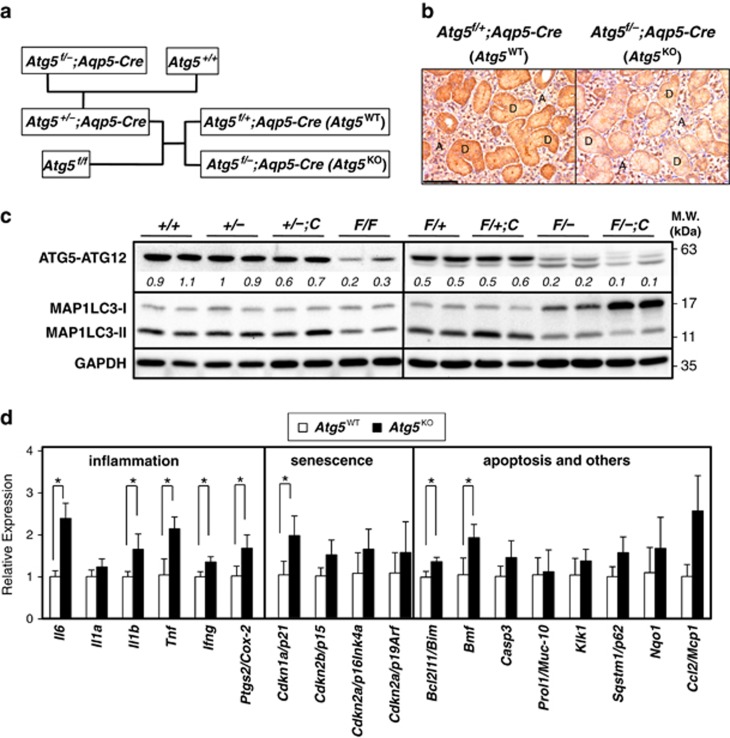
Elevated basal expression of proinflammatory cytokines genes in *Atg5*-knockout SMGs. (**a**) Schematic diagram of generation of experimental mice. Mice developed a spontaneous heterologous *Atg5* deletion after five generations of crossing between *Atg5*^f/f^ and *Atg5*^f/f^;*Aqp5*-Cre mice, resulting in *Atg5*^f/−^;*Aqp5*-Cre mice. *Atg5*^f/+^;*Aqp5*-Cre (*Atg5*^WT^) and *Atg5*^f/−^;*Aqp5*-Cre (*Atg5*^KO^) mice were used in studies herein. (**b**) Immunohistochemical analyses show decreased ATG5 protein in both SMG granular convoluted ducts (GCDs; labeled D) and acinar cells (labeled A) of *Atg5*^KO^ mice, compared with that of *Atg5*^*WT*^ *mice.* Bar: 100 *μ*m. (**c**) Correlation of decreased ATG5 expression and impaired MAP1LC3 lipidation in SMGs among different genotypes. Equal amounts of whole SMG lysates from two individual mouse of the indicated genotype were analyzed for ATG5 and MAP1LC3 levels by western blots. ATG5 expression was greatly reduced in the SMGs of *Atg5*^F/F^ than that in *Atg5*^+/+^ mice. This hypomorphic phenotype in floxed mouse line has been reported previously in the *loxP* mouse gene targeting system.^18^ *+/+*, *Atg5*^+/+^; *+/−*, *Atg5*^+/*−*^; *+/−C*, *Atg5*^+/*−*^;*Aqp5-Cre*; *F/F*, *Atg5*^F/F^; *F/+*, *Atg5*^F/+^; *F/+C*, *Atg5*^F/+^;*Aqp5-Cre* (or *Atg5*^WT^); *F/−*, *Atg5*^F/*−*^; *F/−C*, *Atg5*^F/*−*^;*Aqp5-Cre* (or *Atg5*^KO^). Relative ATG5 levels were determined by setting average level of ATG5 in *Atg5*^+/+^ mice as 1. (**d**) Quantitative RT-PCR analyses show elevated basal expression of selected proinflammatory cytokine genes in SMGs from *Atg5*^*KO*^ *mice.* Respective mean expression level of the indicated message from SMGs of *Atg5*^WT^ mice was set as 1. Results are shown as mean±S.D.; *N*=3; *P<0.05

To assess the contribution of autophagy to tissue injury, we impaired *Atg5* expression in salivary acinar cells by crossing mice expressing aquaporin 5 (*Aqp5*)-driven Cre recombinase^17^ with mice containing *loxP* sites that flank the third exon of *Atg5*^4^ (Figure 1a) and subjected these mice to ductal ligation. Expression of the *Aqp5*-*Cre* transgene mirrored endogenous *Aqp5* in the salivary glands and was acinar cell specific.^17^ Immunohistochemical (IHC) analyses revealed that ATG5 expression was not only abolished in AQP5-expressing acinar cells, but also decreased substantially in AQP5 non-expressors, mainly granular convoluted ducts (GCDs) and other duct cells in the SMGs of *Atg5*^*f/−*^*;Aqp5-Cre* mice (Figure 1b). This is because offspring from *Atg5*^*+/−*^*;Aqp5-Cre* and *Atg5*^*f/f*^ crossbreeds exhibited an ATG5 hypomorphic phenotype in SMGs (Figure 1c, *+/+* *versus* *F/F*) introduced by the flox allele.^18^ As expected, higher ATG5-ATG12 signals were co-existed with higher abundance of lipidated microtubule-associated protein 1 light chain 3 (MAP1LC3-II) (and lower MAP1LC3-I) in the SMGs of *Atg5*^*+/+*^ mice compared with those of *Atg5*^*F/F*^, *Atg5*^*F/−*^ or *Atg5*^*F/−*^*;Aqp5-Cre* mice (Figure 1c). In the ensuing studies, *Atg5*^*f/+*^*;Aqp5-Cre* mice (designated as *Atg5*^WT^) and acinar cell-specific *Atg5*-knockout mice, *Atg5*^*f/−*^*;Aqp5-Cre* (designated as *Atg5*^KO^) were used to simultaneously explore the effects of ATG5 deficiency in acinar cells and reduced ATG5 level in duct cells of SMGs. To document the effect of compromised autophagy on basal gene expression in SMGs, quantitative RT-PCR analyses on message abundance from selected injury response and other genes were performed. Notably, the expression of the proinflammatory mediators such as *Il6*, *Il1b, Tnf* and *Ifng* was significantly elevated in SMGs of KO mice (Figure 1d).

### SMG duct ligation induces autophagy

**Figure 2:**
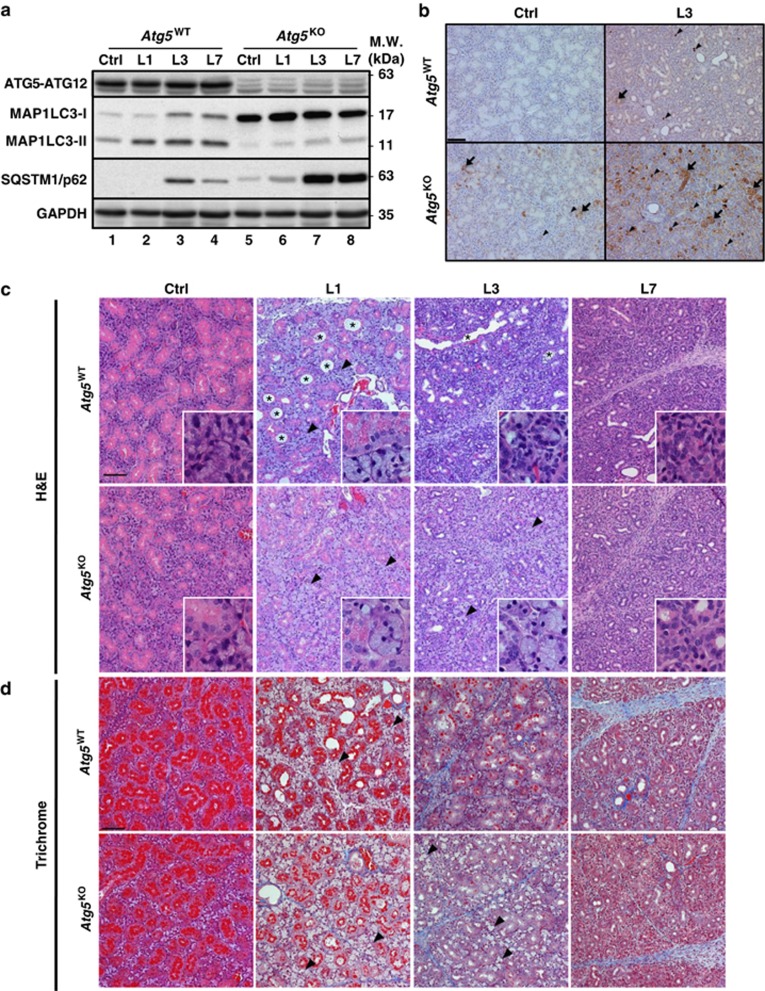
Duct ligation-induced autophagy affects morphological manifestations in post-ligated SMGs. (**a**) Analyses of autophagy-related proteins in post-ligated SMGs from *Atg5*^*WT*^ and *Atg5*^*KO*^ mice. Main excretory ducts from right SMG of individual mouse were ligated for 0 day (control; Ctrl), 1 day (L1), 3 days (L3) or 7 days (L7) before tissue harvesting. Equal amounts of whole gland homogenates were analyzed by western blot using the indicated primary antibodies. One representative western blot is shown (*N*=3). (**b**) Immunohistochemical analyses show intense staining of SQSTM1 in acinar cells (arrowhead) of L3 SMGs from *Atg5*^*KO*^ mice. p62 accumulation was scattered in duct cells (arrow) of ligated glands of *Atg5*^WT^ and control SMGs of *Atg5*^KO^ mice, and became prevalent in both acinar and duct cells of post-ligated L3 SMGs from *Atg5*^KO^ mice. Bar: 100 *μ*m. (**c**) Autophagy impinges on morphological changes induced by duct ligation. SMGs were dissected from *Atg5*^WT^ and *Atg5*^KO^ mice at day 0, day 1, day 3 and day 7 post ligation. Duct obstruction led to gradually reduced eosinophilic secretory granules (bright pink) from GCDs in SMGs of both genotypes, and GCDs were severely dilated (asterisk), especially in L1 SMGs of *Atg5*^WT^ mice. Acinar cells appeared enlarged in L1 SMGs of both genotypes. The enlarged acinar cells persisted in L3 SMGs of *Atg5*^KO^ (arrowhead), but not *Atg5*^WT^ mice, and very few morphologically typical acinar cells were seen in L7 SMGs of both genotypes. Ctrl: non-operated control SMG. Magnification: × 100, and enlarged view (*inset*): × 400. Bar: 100 *μ*m. (**d**) Progression of tissue fibrosis in post-ligated SMGs. FFPE SMG sections were stained with Trichrome. Excess fibrous connective tissues (blue) were present in L3 and L7 SMGs of *Atg5*^WT^ and *Atg5*^KO^ mice. Note the lack of eosinophilic cytoplasmic staining, likely reflecting mucin-like glycoprotein products, in enlarged acinar cells (arrowhead) from L1 glands of both genotypes, and from L3 SMGs of *Atg5*^KO^ mice only. Bar: 100 *μ*m

Next, we investigated whether tissue injury by ligating SMG main excretory ducts from *Atg5*^WT^ and *Atg5*^KO^mice for 1, 3 and 7 days induced autophagy. As expected, the MAP1LC3-interacting sequestosome 1/p62 (SQSTM1), a cargo-recognition protein for autophagy was readily detectable in control SMGs of *Atg5*^KO^, but not *Atg5*^WT^ mice (Figure 2a, *lane 5 versus lane 1*). During ligation, MAP1LC3-II levels increased in *Atg5*^WT^ SMGs at day 1 and remained high throughout the time course (Figure 2a,Supplementary Figure S1A). Additionally, steady-state levels of SQSTM1 were undetectable in control and duct-ligated *Atg5*^WT^ SMGs at day 1, but increased at day 3 (Figure 2a,Supplementary Figure S1B). Notably, SQSTM1 accumulated extensively in the ligated glands from *Atg5*^KO^ mice (Figure 2b,Supplementary Figure S1C). On the basis of comparable *Sqstm1* mRNA levels between these two genotypes, both basal and post-ligation (Figure 1d,Supplementary Figure S2), and sustained accumulation of SQSTM1 protein in ligated SMGs of *Atg5*^KO^mice, we conclude that duct ligation induced autophagy in SMGs.

As depleting ATG5 affected basal abundance of proinflammatory response messages (Figure 1d), the expression of selected inflammatory mediators, including proinflammatory cytokines, *Il6*, *Il1a*, *Il1b*, *Tnf* and *Ifng*, and prostaglandin-endoperoxide synthase 2/cyclooxygenase 2 (*Ptgs2*/*Cox-2*) in the post-ligated SMGs of *Atg5*^WT^ and *Atg5*^KO^ mice was assessed. We found that duct ligation caused robust increases in these mRNA abundances as early as day 1 post ligation in both genotypes, and the inactivation of ATG5 only affected relative Bcl2 modifying factor (*Bmf*) message induction in the ligated SMGs of *Atg5*^KO^ mice compared with *Atg5*^WT^ mice after 1 day of ligation (Supplementary Figure S2). There was an over 300-fold increase in *Il6* mRNA abundance in duct-ligated SMGs of *Atg5*^KO^ mice at day 1, followed by a decline. Similar patterns of *Il1a*, *Il1b* and *Ptgs2/Cox-2* mRNAs surges of different magnitudes were observed in ligated SMGs of *Atg5*^KO^ mice. To validate induction of proinflammatory cytokines, we measured TNF-*α* protein level by enzyme-linked immunosorbent assay (ELISA). TNF-*α* level peaked in L3 SMG from both genotypes at approximately 1.5 ng/mg total protein.

### Autophagy alters duct ligation-induced morphological manifestations

Gross examination revealed that SMG size of *Atg5*^KO^ mice increased initially at day 1, which could be due to a combination of obstructed saliva outflow and an inflammatory response then leveled off at day 3 and atrophied at day 7 after duct ligation (Supplementary Figure S3). In comparison, significant glandular weight loss was detected in ligated SMGs of *Atg5*^WT^ mice at day 3 post ligation (Supplementary Figure S3I). On average, we observed 19% decrease in gland weight compared with the contralateral ligation control SMG in the same mouse, and the degree of atrophy in terms of weight loss was indistinguishable between *Atg5*^WT^ and *Atg5*^KO^ mice at day 7 post ligation (Supplementary Figure S3I). However, gland weights of *Atg5*^KO^ mice were slightly higher than that of *Atg5*^WT^ mice at day 3 post ligation. Microscopically, GCD cells lost many secretory granules and exhibited a dilated phenotype after ligation (Figure 2c). Extensive dilation of ductal system, presumably overloaded with saliva as a result of the outflow obstruction, was particular pronounced in L1 and L3 SMGs of *Atg5*^WT^ mice and to a lesser extent in *Atg5*^KO^ mice (Figure 2c). The level of duct dilation reduced slowly toward baseline from day 3 to day 7 post ligation. In parallel, secretory acinar cells became enlarged and were staining pale from lacking eosinophilic cytoplasmic staining at day 1 in both genotypes (Figure 2c, *L1 inset*). By day 3, enlarged acinar cells remained in SMGs of *Atg5*^KO^ mice (Figure 2c, *L3, arrowheads*); however, they were relatively sparse in the L3 SMGs of *Atg5*^WT^ mice (Supplementary Figure S4A). By day 7, the atrophied glands from both *Atg5*^WT^ and *Atg5*^KO^ mice had residual GCD structures and scattered acinar cells (Figure 2c,Supplementary Figure S4A). Furthermore, in agreement with the observation of robust inflammatory responses (Supplementary Figure S1), trichrome staining revealed extensive tissue fibrosis in SMGs following prolonged ligation (Figure 2d,Supplementary Figure S4B). Mucin-like material (non-eosinophilic staining) accumulated in acinar cells of L1 SMGs from both genotypes and L3 SMGs from *Atg5*^KO^ mice only, but not *Atg5*^WT^ mice. Similar to H & E staining, trichrome staining revealed more severe fibrosis in L3 SMGs of *Atg5*^WT^ mice than the corresponding SMGs of *Atg5*^KO^ mice (Figure 2d,Supplementary Figure S4B).

**Figure 3:**
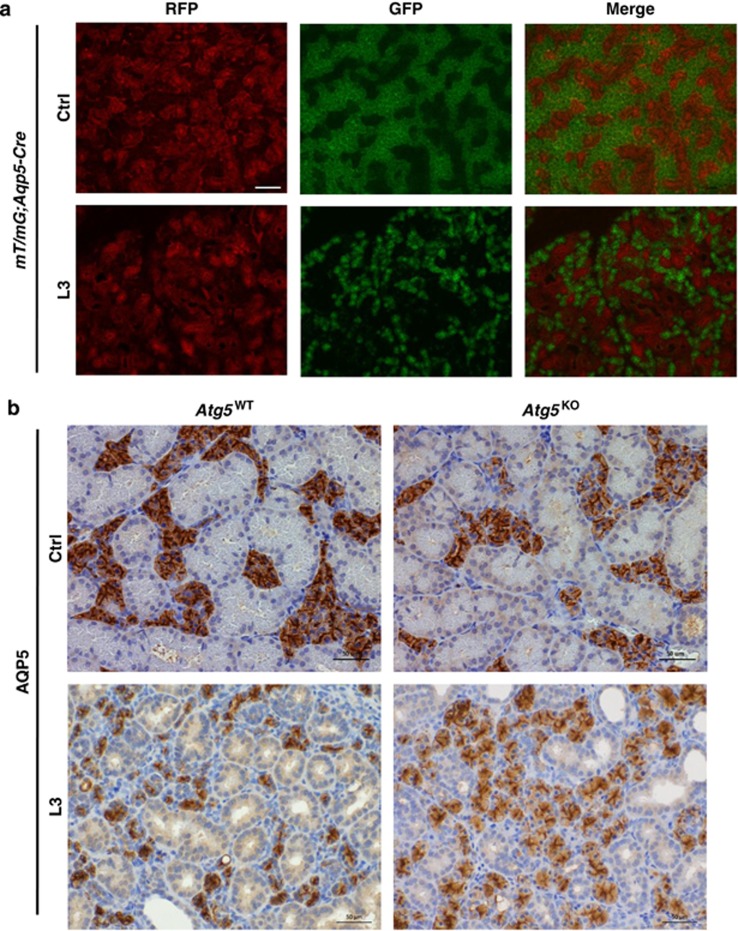
ATG5 exacerbates duct ligation-induced acinar cell atrophy. (**a**) Duct ligation induces acinar cell atrophy. Frozen sections of L3 SMGs and their contralateral counterparts (Ctrl) from *mT/mG;Aqp5-Cre* mice were visualized with fluorescence microscopy. Red fluorescence protein (RFP) was detected ubiquitously in all cells of mT/mG mice except for *Aqp5* promoter-driven *Cre*-expressing acinar cells, which were marked by green fluorescence protein (GFP). Number of GFP-labeled acinar cells was considerably less in L3 gland than control gland. Bar: 100 *μ*m. (**b**) *Suppression of ligation-induced acinar cell death in Atg5*^*KO*^ *mice.* L3 FFPE SMG sections from *Atg5*^WT^ and *Atg5*^KO^ mice were immunostained for acinar-specific AQP5 protein. Number of AQP5-stained acinar cells was considerably less in L3 SMGs of *Atg5*^WT^ than that of *Atg5*^KO^ mice. Bar: 50 *μ*m

To confirm the loss of acinar cells in duct-ligated L3 SMGs of *Atg5*^WT^ mice, we examined red and green fluorescence patterns in duct-ligated SMGs of mT/mG;*Aqp5*-*Cre* reporter mice (Figure 3a). As expected, acinar cells had a distinct pattern of green mG fluorescence due to excision of *loxP*-flanked red mT through acinar-specific *Cre* recombinase, whereas GCDs and other non-acinar cells were mostly marked with red fluorescence in the control gland (Figure 3a, *Ctrl*). Both the size and number of green fluorescent-marked acinar cells in duct-ligated SMGs of mT/mG;*Aqp5*-*Cre* mice were notably reduced at day 3 post ligation (Figure 3a). Additionally, AQP5 IHC staining revealed reduced number of AQP5-positive acinar cells in L3 SMGs of *Atg5*^WT^ mice compared with L3 SMGs of *Atg5*^KO^ mice (Figure 3b).

### ATG5 KO delays ligation-induced apoptosis in SMG acinar cells

**Figure 4:**
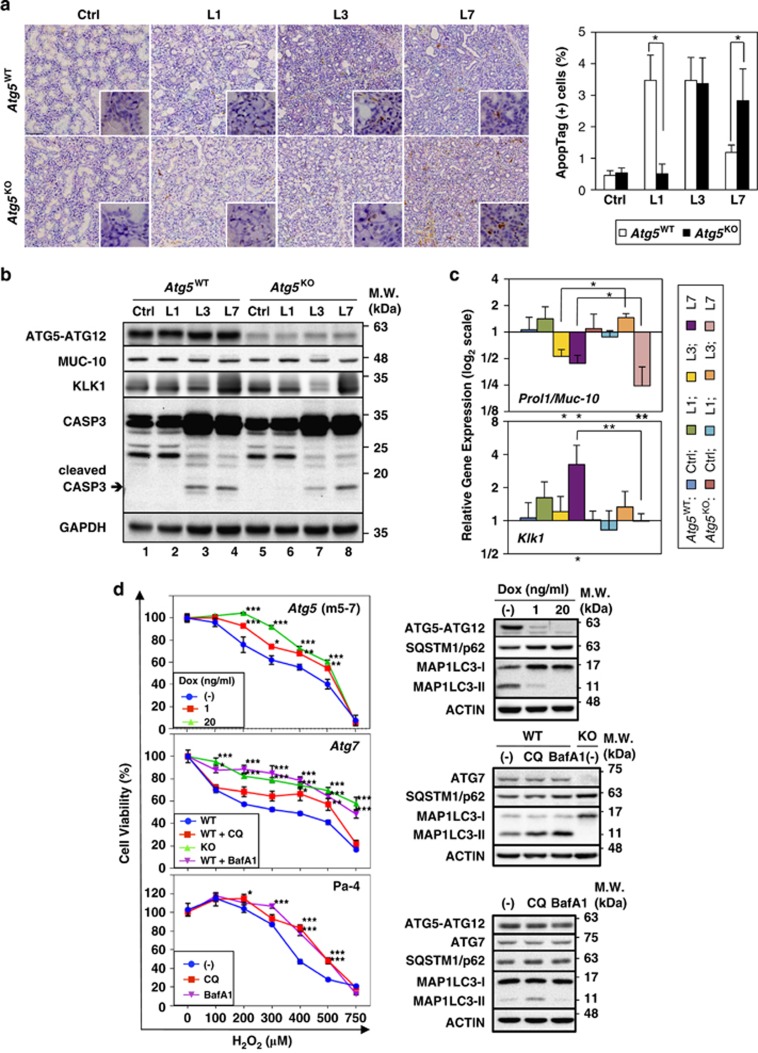
Acinar cell apoptosis is delayed in post-ligated SMGs of ATG5-deficient mice. (**a**) ATG5 status impinges upon duct ligation-triggered acinar apoptosis. Apoptosis by ApopTag Peroxidase (brown nuclear staining) was visualized using an *In situ* Apoptosis Detection Kit (*left panel*). Peak apoptosis was detected in L1 and L3 SMGs of *Atg5*^WT^ mice, whereas the strongest ApopTag signals were detected in L3 and L7 SMGs of *Atg5*^KO^ mice. Magnification: × 100, and enlarged view (*inset*): × 400. Bar: 100 *μ*m. Percent ApopTag-positive cells quantified by dividing the total ApopTag-positive cells by total number of cells examined from 10 randomly chosen fields are shown (*right panel*). Student's *t*-test was employed to determine statistically significant differences in percentage of ApopTag-positive cells between *Atg5*^WT^ and *Atg5*^KO^ groups. Results are shown as mean±S.D.; *P<0.05. (**b**) ATG5 deficiency delays caspase-3 activation. Equal amounts of whole SMG lysates from Ctrl, L1, L3 and L7 SMGs of *Atg5*^WT^ and *Atg5*^KO^mice were analyzed on western blots using indicated antibodies. The cleavage of caspase-3 represents caspase-3 activation. (**c**) Gene expression analyses of *Prol1/Muc-10* and *Klk1*, markers for acinar and duct cells, respectively. SMG duct ligation was performed as described in Figure 2. Expression of the indicated messages in ligated and control SMGs was analyzed by quantitative RT-PCR analyses. Relative gene expression was calculated where respective mean value for control SMG set to 1 for each amplification (*N*≥4). Non-parametric Mann–Whitney test was performed to compare expression levels between respective ligated and control SMGs (_*_ below bars), and between same-day ligated SMGs and control SMGs from *Atg5*^WT^ and *Atg5*^KO^ (_*_ above bars). Results are shown as mean±S.D.; *P<0.05; **P<0.01. (**d**) Autophagy inhibition correlates with resistance to H_2_O_2_-induced cell death. Cell viability was measured in m5-7, *Atg7*-KO MEF, WT MEF and salivary Pa-4 cells treated with indicated concentrations of H_2_O_2_ in combination with chloroquine (CQ; 20 *μ*M) or baflomycin A1 (BafA1; 10 nM) for 48 h and compared with that of the control cells. Data were analyzed with ANOVA followed by Bonferroni *t*-test to determine the statistical differences between treatment and control group (*left panels*). Representative western blots (*right panels*), in which total lysates from cells treated for 6 h with vehicle, CQ (20 *μ*M) or BafA1 (10 nM) were stained with indicated antibodies, are shown. Results are shown as mean±S.E.M.; *N*=3; *P<0.05; **P<0.01; ***P<0.001

We next evaluated the functional role of enhanced autophagy in ligation-induced cell death. *In situ* apoptosis assays revealed that the percentage of ApopTag-positive cells in duct-ligated SMGs of *Atg5*^WT^ mice markedly increased at day 1 to day 3, and then decreased at day 7 (Figure 4a). Conversely, the percentage of ApopTag-positive cells peaked at day 3 and day 7 after ligation in *Atg5*^KO^ mice (Figure 4a). Notably, most ApopTag-positive cells were localized outside the ductal system (Figure 4a). In agreement with *in situ* apoptosis data, caspase-3 activation was comparable between L3 and L7 SMGs, whereas cleaved caspase-3 levels markedly increased between L1 and L3 SMGs (Figure 4b,Supplementary Figure S5A). In addition, the message abundances of both death executor caspase-3 (*Casp3*) and apoptotic mediators *Bcl2l11/Bim* and *Bmf* were significantly upregulated following duct ligation and fold induction of *Bmf*, compared with control, were higher in *Atg5*^WT^ than in *Atg5*^KO^ mice after 1 day of ligation (Supplementary Figure S2). A decrease in the abundance of acinar cell proline rich, lacrimal 1/mucin-10 (*Prol1/Muc-10*) message (Figure 4c), but not MUC-10 protein (Figure 4b,Supplementary Figure S5B), was detected in SMGs from *Atg5*^WT^ at day 3 and day 7 after duct ligation. In contrast, the abundance of ductal marker kallikrein 1 (*Klk1*) message was comparable between the two genotypes, except that it significantly increased by approximately 3-fold over the control level in L7 SMGs of *Atg5*^WT^ mice only (Figure 4c). Notably, the KLK1 protein abundance was higher in SMGs from *Atg5*^WT^ than that of *Atg5*^KO^ at day 1 and day 3 after duct ligation (Figure 4b,Supplementary Figure S5C). Since duct cells contribute to the regeneration of damaged exocrine glands,^19,20^ the increased *KLK1* messages in L7 SMGs of *Atg5*^WT^ mice may reflect the initiation of regeneration.^21^

To validate the participation of ATG5 in cellular injury, H_2_O_2_-induced cell death was examined in *Atg5* Tet-Off MEF m5-7 cells^22^ as an *in vitro* model. ATG5 levels in m5-7 cells were regulated by concentration of doxycycline (Dox; Figure 4d) in culture medium. The decreased ATG5 level clearly compromised cellular autophagic capacity, evident by decreased MAP1LC3-II and increased p62 levels. Notably, an inverse ATG5-dose-dependent correlation was observed between ATG5 protein levels and cell viabilities upon H_2_O_2_ treatment (Figure 4d). In addition to *Atg5*-KO cell model, we also tested sensitivity to H_2_O_2_-induced cell death affected by autophagy inhibition through *Atg7*-KO and autophagy inhibitors, chloroquine and bafilomycin A1 (BafA1), respectively, in *Atg7*-KO MEF and salivary Pa-4 cells. Both autophagy-compromised MEFs and salivary Pa-4 cells demonstrated reduced susceptibility to H_2_O_2_-induced cell death (Figure 4d). Taken together, we surmised that autophagy deficiency renders delayed cell death and/or greater apoptotic threshold upon stress.

### Decreased ATG5 primes SMG duct cells for duct ligation-induced senescent phenotypes

**Figure 5:**
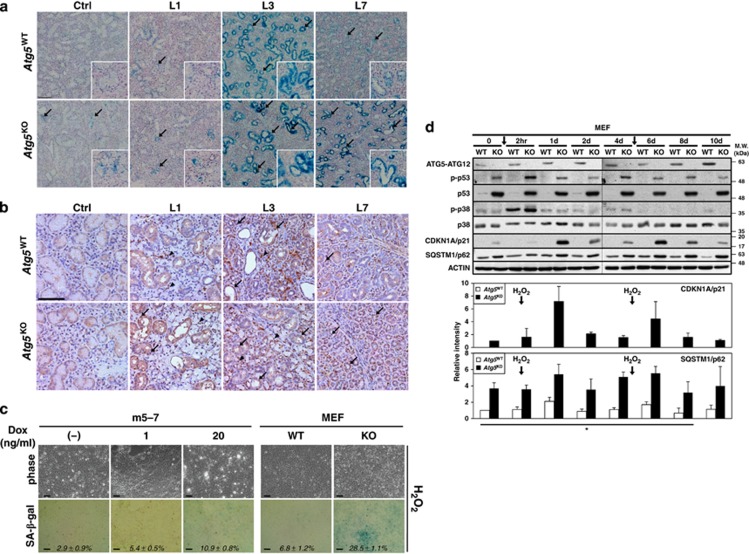
Duct ligation induces sustained premature cellular senescence phenotypes in ATG5-deficient duct cells. (**a**) *ATG5* deficiency predisposes and results in persistent stress-induced SA-β-gal activity in duct cells. Frozen sections of pre-ligated SMGs (Ctrl) of *Atg5*^KO^ mice were positive, albeit weak, for SA-*β*-gal activities (indicated by arrows). Duct ligation intensified SA-*β*-gal activities, which peaked in L3 SMGs from both genotypes. Of note, SA-*β*-gal signals remained strong in L7 SMGs from *Atg5*^KO^ mice while diminished in L7 SMGs from *Atg5*^WT^ mice. Magnification: × 100, and enlarged view (*inset*): 200X. Bar: 100 *μ*m. (**b**) Increased steady-state protein level of senescence marker CDKN1A/p21 in post-ligated glands. FFPE SMG sections were immunostained with an anti-CDKN1A/p21 antibody. Nuclear and cytoplasmic CDKN1A/p21 signals were detected mostly in GCDs (arrow) and some acinar cells (arrowhead) of post-ligated SMGs. Magnification: 200X. Bar: 100 *μ*m. (**c** and **d**) ATG5 level modulates sublethal H_2_O_2_-induced senescent phenotypes in MEF cells. The *Atg5* Tet-Off MEF m5-7 cells, *Atg5*^*−*/*−*^ MEF (KO), and wild-type MEF (WT) cells were treated twice at day 0 and day 5 with *H*_*2*_*O*_*2*_ as described in Materials and Methods, and assayed for SA-*β*-gal activity (**c**) or lysed at different time points and subjected to western analyses with indicated antibodies (**d**). ATG5 expression in *Atg5* Tet-Off MEF m5-7 cell was suppressed by doxycycline (Dox) supplementation, as in Figure 4. Percentages of SA-*β*-gal-positive cells over total number of cells are indicated (*italic*). Treatment with sublethal concentrations of H_2_O_2_ elicited more SA-*β*-gal-positive ATG5-KO MEFs than WT MEFs (**c**). H_2_O_2_ transiently induces p21 in KO MEFs after 1 day of H_2_O_2_ treatment (day 0 and day 5, arrows) (*top panel*) and the quantitative analysis of corresponding p21 and p62 in this representative western is shown below by setting respective p21 protein level of untreated KO MEF ad p62 protein level of untreated WT MEF at one, respectively (*lower panel*) (**d**). Results are shown as mean±S.D.; *N*=3; *P<0.05 (for p62 between two genotypes). Bar: 100 *μ*m

Many upregulated proinflammatory mediator messages in ligated SMGs (Supplementary Figure S2) are also genes encoding SASP factors that are secreted by various cells undergoing premature senescence.^23^ Therefore, we asked whether duct ligation induced cellular senescence. SA-*β*-gal activity, which is strongly associated with senescent cells,^24^ was examined in control and ligated SMGs. While SMG SA-*β*-gal activity was detected sparingly at day 1 following duct ligation, SMGs from both *Atg5*^WT^ and *Atg5*^KO^ mice showed robust SA-*β*-gal staining after 3 days of ligation (Figure 5a, *L3*). Notably, SA-*β*-gal activity was mainly detected in duct cells of GCDs of both genotypes. In addition, SA-*β*-gal activity was detected in limited duct cells of control SMGs from *Atg5*^KO^ mice, but not *Atg5*^WT^ mice. After 7 days of ligation, the SA-*β*-gal staining remained intense in the duct cells of post-ligated SMGs of *Atg5*^KO^ mice, but markedly decreased in the corresponding counterparts of *Atg5*^WT^ mice (Figure 5a, *L7*). We postulated that the attenuated autophagy resulting from reduced ATG5 abundance in SMG duct cells of *Atg5*^KO^ mice (Figure 1b) attributes to the lingering SA-*β*-gal activity from day 3 to day 7 in KO mice.

To corroborate the results of SA-*β*-gal accumulation, we confirmed that other senescence markers, including cell-cycle inhibitors *Cdkn2a/p16Ink4a*, *Cdkn2a/p19Arf*, *Cdkn2b/p15* and *Cdkn1a/p21,*^25^ were upregulated following duct ligation (Supplementary Figure S2). CDKN1A/p21 IHC analyses showed prominent CDKN1A/p21 staining in GCDs of L3 SMGs (Figure 5b), independently validating duct ligation-induced senescent phenotype in duct cells of ligated SMGs. In that, CDKN1A/p21 signal was stronger at day 1 post ligation in *Atg5*^KO^mice than in *Atg5*^WT^ mice. Consistent with SA-*β*-gal activity (Figure 5a), the majority of GCDs in SMGs of *Atg5*^KO^ mice at day 7 post ligation stained positive for CDKN1A/p21, but much less so in L7 SMGs of *Atg5*^WT^ mice (Figure 5b,Supplementary Figure S6).

We then used a cell-based assay to confirm the role of ATG5 in premature senescence by examining H_2_O_2_-induced senescent phenotype in *Atg5* Tet-Off MEF m5-7 and *Atg5*-KO MEF cells.^22^ Treatment with sublethal concentrations of H_2_O_2_ resulted in more SA-*β*-gal-positive *Atg5*-KO MEF cells than in WT MEF cells (Figure 5c). In addition, steady-state abundance of CDKN1A/p21 protein was substantially increased by sublethal concentrations of H_2_O_2_ treatment in *Atg5*-KO MEF cells (Figure 5d). Time course analysis revealed that p21 level peaked in KO but not in WT cells, 1 day after H_2_O_2_ treatment, while p38 was activated immediate (2 h) after the treatment in both cells (Figure 5d). In addition, there is a positive correlation between protein levels of p21 and p62 accumulation (Figure 5d).

### Macrophage activation during duct ligation

Compare with *Atg5*^WT^ mice, duct ligation induced sustained senescent phenotypes in SMG duct cells (Figures 5a and b) and delayed apoptosis in acinar cells (Figures 3 and 4) of *Atg5*^KO^ mice. Macrophages are key components in tissue repair and remodeling during wound healing. To determine whether distinct programs for clearance of damaged cells may have arisen based on cellular autophagy proficiency, we examined recruitment of macrophages following duct ligation. IHC staining for F4/80 revealed that tissue injury induced by duct ligation was accompanied by macrophage recruitment throughout the progression of the tissue injury (Supplementary Figure S7). Notably, F4/80-positive macrophages massively accumulated in injured SMGs at day 3 and day 7 after ligation, however, indistinguishable between WT and KO mice. In addition, expression level of the chemoattractant chemokine chemokine (C-C motif) ligand 2/monocyte chemoattractant protein-1 (*Ccl2/MCP-1*), which was highly induced in SMGs following ligation (Supplementary Figure S2), was also comparable between the two genotypes. We concluded that levels of ATG5 did not affect macrophage activation in the duct ligation model.

## Discussion

**Figure 6:**
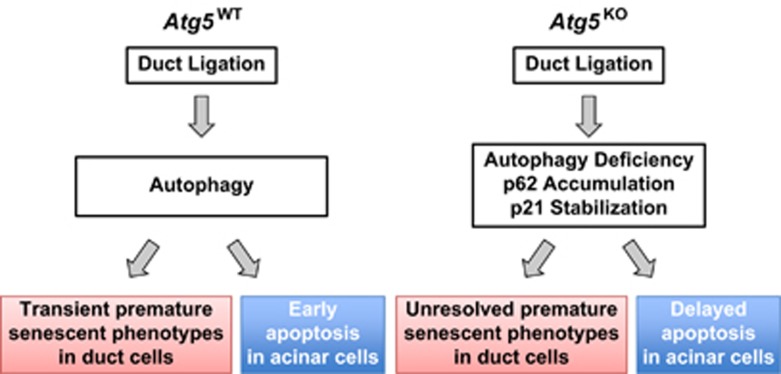
Model for role of ATG5 in ligation-induced apoptosis and premature senescent phenotypes. Duct ligation induces autophagy, acinar cell death through apoptosis, and transient senescent phenotypes in duct cells of SMGs from WT mice. ATG5 deficiency disturbed homeostasis in tissue responses to obstruction stressor stemming from duct ligation, rendering sustained senescent phenotypes in duct cells and delayed apoptotic cell death in acinar cells. The lack of contribution of cleaved ATG5 to apoptosis^34^ could account for the delayed acinar cell death in duct-ligated SMGs of KO mice. On the other hand, sustained senescent phenotypes in duct cells may be due to stabilization of p21 through sequestration by excess polyubiquitin-binding protein p62 in duct-ligated KO mice

It is now generally recognized that autophagic process has a pivotal role in cellular homeostasis and tissue adaptive responses to stress and injury. Herein, we used an injury model of SMG duct obstruction and conditional *Atg5* KO to explore the simultaneous interplay among autophagy, inflammation, stress-induced premature senescence and apoptosis *in vivo*. Ligation of excretory duct of exocrine glands, such as SMG^26^ and pancreas,^27^ induces acinar cell injury and death as well as ductal cell proliferation when the passage of secretions is blocked. However, the mechanism dictating the fate of individual cell types, in response to stress, within the same tissue is still unclear. Our report represents the first of its kind using conditional ATG5-KO mice to delineate molecular mechanism of duct cell survival and acinar cell death in atrophied SMGs following duct ligation *in vivo*. One major finding of this study is that combined effect from reduced ATG5 abundance and disruption of SMG duct ligation-induced autophagy causes a phenotype of delayed apoptosis in acinar cells and sustained premature senescence in duct cells of post-ligated SMGs from *Atg5*^KO^ mice (Figure 6).

Conceivably, depending on stress intensity, autophagy could regulate cell death pathway with two distinct outcomes: (1) the induction of autophagy in responses to ductal obstruction may be initially beneficial for ligated SMGs by eliminating superfluous proteins accumulated within acinar cells due to blocked secretory outflow. This will allow cells to adapt to the hostile conditions. (2) The sustained autophagy may exacerbate stress condition and induce autophagic cell death (or ‘autosis') accompanied by ER dilation and nuclear convolution.^28^ Alternatively, autophagosomes could serve as platforms for caspase activation,^29^ degrading anti-apoptotic factors,^30^ and crosstalking with cell killing mechanisms such as p53 and JNK pathways.^31^ Here we showed that acinar cells in post-ligated SMGs underwent programmed cell death after prolonged ligation. Although apoptosis occurred regardless of autophagy status, apoptotic cell death was delayed in ATG5-deficient acinar cells, compared with autophagy-competent cells (Figures 3 and 4a). Furthermore, autophagy-impaired cells were more resistant to H_2_O_2_-induce cell lethality (Figure 4d), supporting our notion. ATG5, although an essential protein in autophagosome formation, has additional functions beyond autophagy. For instance, both ATG5-dependent mitotic catastrophe^32^ and cell death^33^ in cells treated with cytotoxic agents have recently been reported. More importantly, calpain-cleaved ATG5 is known to activate caspases to enhance susceptibility toward apoptotic stimuli, thus ATG5 functions as a molecular link between autophagy and apoptosis.^34^ Conceivably, the absence of ATG5-dependent caspase activation could account for the initially delayed apoptosis in day-1 post-ligated SMGs of the *Atg5*^KO^ mice. Thus, the delayed *Atg5*-KO acinar cell death could be either autophagy dependent or ATG5 dependent or their combination.

Senescence is a state of stable cell-cycle arrest.^35,36^ Accumulating evidence from several *in vivo* senescent mouse models have suggested that the early onset of senescent phenotypes results from the expression, activation or deletion of genes involved in cell-cycle progression.^37, 38, 39, 40, 41^ Controversy over the relationship between autophagy and senescence exists because both a direct relationship and an inverse relationship were reported.^42^ In agreement with inverse relationships,^9,43^ the emergence of unstressed SA-*β*-gal activity in duct cells of SMGs was only detected from young (6–8 weeks old) *Atg5*^KO^, but not from *Atg5*^WT^, mice (Figure 5a, *Ctrl*). Moreover, stress-induced senescent phenotypes were activated transiently in duct cells of post-ligated SMGs from *Atg5*^WT^ mice, however, persisted through day 7 in *Atg5*^KO^ mice (Figures 5a and b). We hypothesize that senescent phenotypes were transient in duct-ligated SMGs from *Atg5*^WT^ mice because autophagy removes superfluous proteins resulted from blunted secretion, thus maintains cellular homeostasis. When the clearance mechanism is impaired, damaged organelles and other proteins accumulate, leading to unresolved stress phenotypes in the autophagy-impaired SMGs. These observations of increased p21 protein level and intensified SA-*β*-gal signals in sublethal H_2_O_2_-treated *Atg5*-compromised MEF cells than the corresponding WT MEF cells (Figures 5c and d) supported the notion that lower ATG5 abundance in duct cells of *Atg5*^KO^ mice, compared with *Atg5*^WT^ mice (Figure 1b), primed these SMG duct cells susceptibility to persistent senescent-associated phenotypes (Figure 5a).

Extended outflow blockage led to extensive accumulation of p62 protein in SMGs of KO mice (Figures 2a and b). The polyubiquitin-binding protein p62, through interaction with LC3, is involved in delivering cargoes to the autophagy machinery or lysosomes for degradation.^44^ In autophagy-deficient *Atg5*-KO cells, excessive p62, instead, sequesters ubiquitinated proteins from proteasomal degradation, extending their half-life.^45^ Conceivably, p21, a short-lived protein, could therefore prevail in day-7 post-ligated SMGs of *Atg5*^KO^ mice through reduced turnover (Figure 5b). However, we cannot rule out the possibility that the stressed acinar cells might modulate the fate of duct cells in a paracrine and autophagy-dependent manner. Even though cellular senescence is generally considered as irreversible, escape scenarios, such as p53 pathway inactivation, exist to allow cells to re-enter the cell cycle.^13,46^ Duct ligation clearly leads to a transient senescent phenotype in post-ligated duct cells of *Atg5*^WT^ but not *Atg5*^KO^ mice. The exact underlying mechanism is still unclear. It is possible that autophagy keeps p62 levels in check, thus promotes resolution of senescent phenotype. Alternatively, autophagy could facilitate the removal of dead cells at the final stage of post-ligation period, minimizing the stress input from local environment.

Collectively, our results explicitly show that ATG5 deficiency primes SMG cells with increased expression of proinflammatory cytokine and *Cdkn1a/p21* messages, and the lack of proper duct ligation-induced autophagy dysregulates the tempo and duration of sequential and overlapping elements of tissue injury, including inflammatory responses, stress-induced premature senescence and apoptosis. We therefore conclude that duct ligation-induced autophagy has a dynamic role in preserving the structural integrity of duct and acinar cells in duct-obstructed SMGs. One potentially important mechanism of regulating autophagy in post-ligated SMGs is through the mammalian target of rapamycin (mTOR). Bozorgi *et al.*^14^ have recently reported while that mTOR is switched off in normal salivary glands, it gets switched on during the course of duct ligation. As mTOR inhibition is instrumental for autophagy induction,^1^ the activation of mTORC1 appears to be sufficient to suppress autophagy by preventing the formation of ATG complex.^47^ One possible explanation to reconcile our data with theirs is that mTOR activation could represent a feedback mechanism to escape from autosis or autophagy-induced cell death. Nonetheless, these discordances underlie the complexity of *in vivo* role ATG5 or autophagy partakes in post-ligated SMGs and hint at their respective interactions with other stress-triggered signaling pathways.

## Materials and Methods

### Generation of salivary acinar-specific *Atg5*-deficient mice

Mice with salivary acinar-specific ATG5 deficiency were generated by crossing *Atg5*^*flox/flox*^ mice (*Atg5*^*f/f*^)^4^ with *Aqp5*-*Cre* mice^17^ that express Cre recombinase under the control of the *Aqp5* promoter. *Atg5*^*f/−*^;*Aqp5*-*Cre* genotypes arose from breeding *Atg5*^*f/f*^ with *Atg5*^*f/f*^*;Aqp5-Cre* mice for several generations, most likely through promiscuous Cre recombinase activity during developmental processes, such as gametogenesis.^48^ These mice were selected for mating with C57BL/6 mice to generate the *Atg5*^*+/−*^;*Aqp5*-*Cre* mice used in this study. Crossing *Atg5*^*f/f*^ and *Atg5*^*+/−*^;*Aqp5*-*Cre* mice generated *Atg5*^*f/−*^;*Aqp5*-*Cre* (*Atg5*^KO^) and *Atg5*^*f/+*^;*Aqp5*-*Cre* (*Atg5*^WT^) mice. Both *Atg5*^KO^ and *Atg5*^WT^ mice were on a mixed C57BL/6 and 129S6/SvEvTac background and appeared normal without gross abnormality. Genotypes was screened using the following PCR primer pairs: *Atg5*, forward, 5′-AAGCACCTAGTCACACCACATCCA-3′, and reverse, 5′-CACGTGTGAGTGATGGTTGGCTTT-3′ for simultaneously detecting the WT and floxed *Atg5* alleles; Δ*Atg5*; forward, 5′-CAGGGAATGGTGTCTCCCAC-3′, and reverse, 5′-GTACTGCATAATGGTTTAACTCTTGC-3′ to detect deleted the *Atg5* allele; and *Cre*, forward, 5′-TGCCCAAGAAGAAGAGGAAGGTGT-3′, and reverse, 5′-GCCGCATAACCAGTGAAACAGCAT-3′.

### Generation of reporter mice

Reporter mT/mG;*Aqp5*-*Cre* mice were generated by crossing mT/mG^49^ and *Aqp5*-*Cre* mice.^17^

### Cell line, H_2_O_2_ treatment and cell viability assay

WT, *Atg5*-KO and *Atg7*-KO MEF cells (kind gift from Dr. Chengyu Liang, USC) were cultured in DMEM with 10% FBS. Salivary Pa-4 cells were cultured as we described previously.^50^ The *Atg5* Tet-Off MEF m5-7 cells^22^ were maintained in DMEM containing 10% FBS with doxycycline (Dox, 20 ng/ml) to fully suppress *Atg5* expression, as we described previously.^51^ To restore *Atg5* expression, cells were cultured either in Dox-free medium for full restoration or in medium with 1 ng/ml Dox for partial restoration. These cells were treated with up to 1 mM H_2_O_2_ alone or in the presence of chloroquine (20 *μ*M) or BafA1 (10 nM) as indicated, and cell viability was assessed 48 h later based on acid phosphatase assay described by Yang *et al.*^52^ In brief, cells in a 96-well plate were washed with PBS and incubated with 100 *μ*l p-nitrophenyl phosphate (Sigma-Aldrich, St. Louis, MO, USA; N4645) in 5 mM in 0.1 M sodium acetate and 0.1% Triton X-100, pH 5.0, for 1 h at 37 °C. The reaction was stopped with the addition of NaOH (10 *μ*l, 1 N) and absorbance measured at 405 nm using a microplate reader. For senescence induction, m5-7 cells were maintained in growth media containing different concentrations of doxycycline (0, 1 and 20 ng/ml). At day 1 after seeding, cells were treated with sublethal dose of H_2_O_2_ (75 *μ*M) for 2 h, washed and cultured in growth medium for 5 days. The H_2_O_2_ treatment and recovery were repeated once before cell harvesting. WT and *Atg5*-KO MEF cells were treated with 150 *μ*M H_2_O_2_ using the same protocol as for m5-7 cells, and the cells were analyzed for SA-*β*-gal activity as below.

### Fluorescent microscopy

Fluorescent images of frozen sections from SMG of mT/mG;*Aqp5*-*Cre* mice were acquired using an Olympus AX70 microscope (Olympus, Tokyo, Japan). All images were compiled using Image-Pro (version 6.3, Media Cybernetics, Rockville, MD, USA).

### Submandibular duct ligation

Due to gender dimorphism in mouse SMGs,^53^ male mice (6–8 weeks old) were used exclusively in all ligation studies reported here. Mice were maintained under 12-h light/dark cycles with unlimited access to food and water. Unilateral ligation of the main excretory ducts of right SMGs, for a period from 1 to 7 days, was performed using surgical sutures, according to Turner *et al.*^54^ The contralateral left SMGs, which served as ligation controls, from the same mice were also harvested for comparison. Animals were euthanized at indicated times following ligation. Both control and ligated SMGs were removed and either snap-frozen in liquid nitrogen for western and real-time RT-PCR analyses or fixed in 4% paraformaldehyde for Hematoxylin and Eosin (H&E) staining and IHC analyses. City of Hope Institutional Animal Care and Use Committee approved all surgical procedures reported here.

### IHC analysis

IHC staining was performed on 5-*μ*m thick tissue sections prepared from formalin fixed, paraffin-embedded (FFPE) tissues. FFPE tissue sections were deparaffinized in xylene then hydrated through graded ethanol and distilled water. Samples were then quenched in 1% hydrogen peroxide and pretreated with steam from citrate buffer (Vector Laboratories, Burlingame, CA, USA; H-3300) to promote antigen retrieval. After antigen retrieval, slides were incubated in Protein Block for 1 h, then incubated with primary antibodies ATG5 (1 :  800 dilution, RT, 1 h; Novus Biologicals, Littleton, CO, USA; NB110-53818), SQSTM1/p62 (1 : 1500 dilution, 4 °C, overnight; Wako Chemicals, Richmond, VA, USA; 018-22141), AQP5 (1 : 150 dilution, RT, 30 min; Alomone Labs, Jerusalem, Israel; AQP-005), F4/80 (1 : 50 dilution, RT, 30 min; AbD Serotec, Raleigh, NC, USA; MCA497R), or CDKN1 A/p21 (1 : 1000 dilution, 4 °C, overnight; Abcam, Cambridge, MA, USA; ab2961), respectively. For ATG5, SQSTM1 and CDKN1A/p21 IHCs, slides were then washed in PBS and incubated with biotinylated secondary antibody (1 : 200 dilution, RT, 30 min; Vector Labs, BA-1000) and VECTASTAIN Elite ABC protocol followed (Vector Laboratories, PK-6100). For AQP5 and F4/80 IHCs, slides were washed in Dako buffer after primary antibody incubation and incubated with EnVision+ (Dako, Carpinteria, CA, USA; K4010) secondary antibody for 30 min. After three more washes in Dako buffer, slides were incubated with chromogen 3,3′-diaminobenzidine tetrahydrochloride (DAB), counterstained with hematoxylin, and mounted.

### Real-time reverse-transcription PCR

Total RNA was extracted from SMGs with TRIzol (Life Technologies, Grand Island, NY, USA), according to the manufacturer's instructions. Synthesis of cDNA was performed using the iScript cDNA Synthesis Kit (Bio-Rad, Hercules, CA, USA; 170-8891). The cDNA was amplified using IQ SYBR Green Supermix and specific primer pairs in a My IQ Real-Time PCR Detection System (Bio-Rad, 170-8882). Relative mRNA expression levels were calculated using the ΔC_t_ method, as previously described,^50^ against *Gclc*, a message that we found stable throughout the duct ligation process. The primer pairs used are shown in Supplementary Table S1.

### Western analyses

Whole tissue lysates were prepared using the Qproteome Mammalian Protein Prep Kit (Qiagen, Valencia, CA, USA; 37901) according to the manufacturer's protocol, and then supplemented with Complete Protease Inhibitor Cocktail (Roche, Indianapolis, IN, USA). Equal amounts of tissue or cell lysates were separated on SDS-PAGE and then immunoblotted with antibodies that recognize ATG5 (Novus Biologicals, NB110-53818), ATG7 (Cell Signaling, Danvers, MA, USA; 2631), SQSTM1/p62 (American Research Products, Waltham, MA, USA; 03-GP62-C), MAP1LC3 (Nanotools, München, Germany; 0231-100/LC3-5F10), CDKN1A/p21 (Santa Cruz Biotechnology, Dallas, TX, USA; sc-397) MUCIN10 (MyBiosource, San Diego, CA, USA; MBS422856), KALLIKREIN1 (Boster Biological, Pleasanton, CA, USA; PA1709), CASPASE-3 (Cell Signaling, 9665), p53 (Santa Cruz Biotechnology, sc-6243), phospho-p53 (Cell Signaling, 9284), p38 (Cell Signaling, 9212), phospho-p38 (Cell Signaling, 9211), Actin (EMD Millipore, Billerica, MA, USA; MAB1501R), and GAPDH (Santa Cruz Biotechnology, sc-25778). Blots were visualized using an enhanced chemiluminescence detection kit (ECL-Plus, GE Healthcare, Pittsburgh, PA, USA; RPN2132) and a VersaDoc 5000 Imaging System (Bio-Rad) as we described previously.^50^ Signal intensities of the captured images were analyzed with the Quantity One Software (Bio-Rad). Results of western blots shown are representatives of two to four independent experiments.

### Quantitation of TNF-*α*

Tissue lysates were diluted ten-fold with 1X Assay Diluent B and levels of TNF-*α* were determined by the TNF alpha Mouse ELISA Kit (Abcam, ab100747) following the manufacturer's protocol.

### SA-*β*-gal Staining

Control and duct-ligated SMGs were embedded in OCT compound for cryosection and stained for SA-*β*-gal activity according to the manufacturer's protocol (Cell Signaling Technology, 9860). Tissue slides were counterstained with hematoxylin before mounting medium was applied. The H_2_O_2_-treated m5-7 and MEF cells were fixed in 2% fomaldehyde, 0.2% glutaraldehyde in PBS for 10 min at room temperature and stained for SA-*β*-gal activity as above.

### *In situ* apoptosis detection

Apoptosis was detected in FFPE tissue slides (prepared as described above) using an ApopTag Peroxidase *In Situ* Apoptosis Detection Kit (EMD Millipore, S7100) following the manufacturer's instructions. The percentage of apoptotic cells was calculated by dividing the number of ApopTag-positive cells over total number of nuclei enumerated from randomly selected ten microscopic fields (10X) of individual samples using ImagePro.

### Statistical analysis

Gene expression data from quantitative RT-PCR were processed by Microsoft Excel, and analyzed by using GraphPad Prism 6.0 (GraphPad Software, La Jolla, CA, USA). The non-parametric Mann–Whitney test was employed to determine the significant differences between the ligated groups and control group of each genotype as well as the significant differences between *Atg5*^WT^ and *Atg5*^KO^ mice. Statistic significance of *in situ* apoptosis assays between *Atg5*^WT^ and *Atg5*^KO^ groups was determined using Student's *t*-test. For cell viability assay, data were analyzed with ANOVA followed by Bonferroni *t*-test.

## Abbreviations

AQP5: aquaporin 5
ATG5: autophagy-related 5
ATG7: autophagy-related 7
BafA1: bafilomycin A1
*Bcl2l11/Bim*: Bcl2-like 11
*Bmf*: Bcl2 modifying factor
*Casp3*: caspase-3
*Ccl2/MCP-1*: chemokine (C-C motif) ligand 2/monocyte chemoattractant protein-1
*Cdkn*: cyclin-dependent kinase inhibitor
CQ: chloroquine
ELISA: enzyme-linked immunosorbent assay
*Gclc*: glutamate-cysteine ligase, catalytic subunit
GCD: granular convoluted duct
GFP: green fluorescent protein
*Il1*: interleukin 1
*Il6*: interleukin 6
*Ifng*: interferon gamma
KO: knockout
*Klk1*: kallikrein 1
MAP1LC3: microtubule-associated protein 1 light chain 3
*Nqo1*: NADPH dehydrogenase, quinone 1
*Prol1/Muc-10*: proline rich, lacrimal 1/mucin-10
*Ptgs2/Cox-2*: prostaglandin-endoperoxide synthase 2/cyclooxygenase 2
RFP: red fluorescent protein
*Sqstm1*/*p62*: sequestosome 1
SA-*β*-gal: senescence-associated *β*-galactosidase
SASP: senescence-associated secretory phenotype
SMG: submandibular gland
*Tnf*: tumor necrosis factor
WT: wild type
