# Supplementary material for: Dynamic involvement of ATG5 in cellular stress responses
Source: Cell Death Dis. 2014 Oct 23;5(10):e1478–. doi: 10.1038/cddis.2014.428 (PMC4649523; doi:10.1038/cddis.2014.428)
Supplement: Supplementary Information [file cddis2014428x1.doc]

**Supplemental Table S1: Sequences of Quantitative RT-PCR Primer Pairs**

| Gene Name | Forward Primer | Reverse Primer |
| --- | --- | --- |
| *Bcl2l11/Bim* | GGTATGGAGAAGGCATTGACAGG | CTCTGGGATAGAACCACTGGACAC |
| *Bmf* | ACTACTGCCACCTTTGCTGGAG | TGCTTCTGCTGAGATGGATGC |
| *Casp3* | tggtgatgaaggggtcatttatg | ttcggctttccagtcagactc |
| *Ccl2/MCP-1* | GTGCTGACCCCAAGAAGGAA | GTGCTGAAGACCTTAGGGCA |
| *Cdkn1a/p21* | AGGAGCAAAGTGTGCCGTTG | CGAAGTCAAAGTTCCACCGTTC |
| *Cdkn2a/p16Ink4a* | TTTCGGTCGTACCCCGATTC | TGCACCGTAGTTGAGCAGAAGAG |
| *Cdkn2a/p19Arf* | TGGTGAAGTTCGTGCGATCC | CCCTCTTCTCAAGATCCTCTCTAGC |
| *Cdkn2b/p15* | AGATCCCAACGCCCTGAACC | ACAGGTCTGGTAAGGGTGGC |
| *Gclc* | CTCTGCCTATGTGGTATTCGTGG | GCAAGACAGCATCTCGCTTCTG |
| *Ifng* | CTTGAACCCTGTCGTATGCTGG | TTGGTGCAGGAATCAGTCCAGG |
| *Il1a* | TGTTGCTGAAGGAGTTGCCAG | CCCGACTTTGTTCTTTGGTGG |
| *Il1b* | TGGACCTTCCAGGATGAGGACA | GTTCATCTCGGAGCCTGTAGTG |
| *Il6* | TACCACTTCACAAGTCGGAGGC | CTGCAAGTGCATCATCGTTGTTC |
| *Klk1* | GGTGTGGCTGGGCAAAAA | TTGTGGGGTGTGCTCATTCAG |
| *Nqo1* | CTCAACTGGTTTACAGCATTGGC | TGGAGCAAAATAGAGTGGGGTCTC |
| *Prol1/Muc-10* | CCACCAAATCTACAACAAAAGAACC | TCCAAAACACTTCCGCAAATG |
| *Ptgs2/Cox-2* | TCCAACCTCTCCTACTACACCAGG | GCTCCTTATTTCCCTTCACACCC |
| *Sqstm1/p62* | ATGGGTTTCTCGGATGAAGGCG | CTATCACAATGGTGGAGGGTGC |
| *Tnf* | GTTCATCCATTCTCTACCCAGCC | TGTCCCAGCATCTTGTGTTTCTG |

**Supplemental Figure Legends**

**Supplemental Fig. S1. Duct ligation induces p62/SQSTM1 accumulation in SMGs of both genotypes.** Quantified bar graph of relative protein LC3-II to LC3-I ratio **(A)** and p62 abundance **(B)** after normalization with GAPDH, as shown in Fig. 2A. Results are shown as mean ± S.D.; N = 3. (**C**) Quantitative analysis of p62-positive acinar and ductal cells, respectively, from ten randomly selected microscopic fields (10X) from respective panels in Fig. 2B using ImagePro. The percentage of p62-positive cells with indicated acinar or ductal morphologies was determined by dividing by total number of respective enumerated cells. *: *p* < 0.05: **: *p* < 0.01: ***: *p* < 0.001.

**Supplemental Fig. S2. Duct ligation induces marked increases selected mRNA abundances over control levels.** Duct ligation of SMGs from *Atg5*^WT^ and *Atg5*^KO^ mice for a period of 0-, 1-, 3- or 7-days was performed as described in Fig. 2. Message levels of the selected genes in harvested SMGs were analyzed by quantitative RT-PCR by designating the respective expression level of individual genes in SMGs of both *Atg5*^WT^ and *Atg5*^KO^ control mice (Ctrl) as 1. Relative expression levels of mRNA were consistent among individual mice within the control groups. However, post-ligation gene expression profiles were more variable, likely resulted from differing injury responses to ligation from individual mouse. Non-parametric Mann-Whitney test comparing expression levels between ligated and respective control SMGs (* below bars), and between same-day ligated SMGs from *Atg5*^WT^ and *Atg5*^KO^ mice (* above bars) was performed. Results are shown as mean ± S.D.; N ≥ 4; *: *p* < 0.05; **: *p* < 0.01.

**Supplemental Fig. S3. Pronounced morphological manifestations in post-ligated SMGs from *Atg5*^WT^ mice. (A-H)** Whole gland FFPE slices (5 μm thick) were stained with H&E and examined under an Olympus AX70 microscope using 4X objectives. The images shown were assembled from a series of 2 x 3 (4X) to 3 x 4 (4X) tiles using Image-Pro (version 6.3). Duct-ligation induces severe glandular atrophy after 7 days in L7 SMGs from both *Atg5*^WT^ and *Atg5*^KO^ mice. Numerous unstained, small circular- and convoluted-shaped and dilated GCDs were visible in parenchyma of SMGs, and were especially pronounced in L1 and L3 post-ligated SMGs from *Atg5*^WT^ mice. SMG; submandibular gland, SLG; sublingual gland. Bar: 500 μm. **(I)** Significant difference in gland weight was observed at day-3 post-ligation between two genotypes. Bar graph also shows changes in SMG gland weight following duct ligation between ligated and respective control SMGs (* below bars). Mann-Whitney Nonparametric test was used for statistical analyses. Results are shown as mean ± S.D.; N ≥ 4; *: *p* < 0.05.

**Supplemental Fig. S4. Quantitative analyses of duct ligation-induced morphological manifestations in post-ligated SMGs.** (**A**) Quantitative analysis of relative acinar cell number remaining following ligation, as shown in Fig. 2C. The percentage of acinar cells was calculated by dividing the number of acinar cells over the number of acinar cells in unligated SMGs from *Atg5*^WT^ mice from ten randomly selected microscopic fields (10X) of respective panels in Fig. 2C using ImagePro. (**B**) Area of fibrosis was quantified from images of trichrome-stained tissue slides, as shown in Fig. 2D, with method described by Haller, S.T. et al. , using Image J (version 1.49c) software (NIH). Results are shown as mean ± S.D.; N ≥ 4; *: p < 0.05; **: p < 0.01; ***: p < 0.001.

**Supplemental Fig. S5. Quantitative densitometric analysis of cleaved caspase-3, MUC-10 and KLK1 protein in post-ligation SMGs.** Bar graph shows the percentage of cleaved caspase-3 over caspase-3 (**A**), and relative steady-state protein abundance of MUC-10 (**B**) and KLK1 (**C**) after normalization with GAPDH, as shown in Fig 4B. Results are shown as mean ± S.D.; N = 3; *: *p* < 0.05.

**Supplemental Fig. S6. Quantitative analysis of p21-positive cells in post-ligated SMGs.** The percentage of p21-positive cells was calculated by dividing the number of p21-positive cells over total number of nuclei enumerated from ten randomly selected microscopic fields (10X) of individual samples, as shown in Fig. 5B, using ImagePro. *: *p* < 0.05.

**Supplemental Fig. S7. Macrophages are activated in ligated SMGs of both *Atg5*^WT^ and *Atg5*^KO^ mice.** IHC staining of F4/80 shows that activated macrophages are present throughout the areas of tissue injury induced by duct ligation. The presence of F4/80-positive macrophages (arrow) was not affected by autophagy status. In comparison, the enlarged acinar cells (arrowhead) persisted in L3 SMGs of *Atg5*^KO^, but not *Atg5*^WT^ mice. Bar: 50 μm.
