# Supplementary material for: Dynamic involvement of ATG5 in cellular stress responses
Source: Cell Death Dis. 2014 Oct 23;5(10):e1478–. doi: 10.1038/cddis.2014.428 (PMC4649523; doi:10.1038/cddis.2014.428)
Supplement: Supplementary Figure S1 [file cddis2014428x2.ppt]

## Slide 1
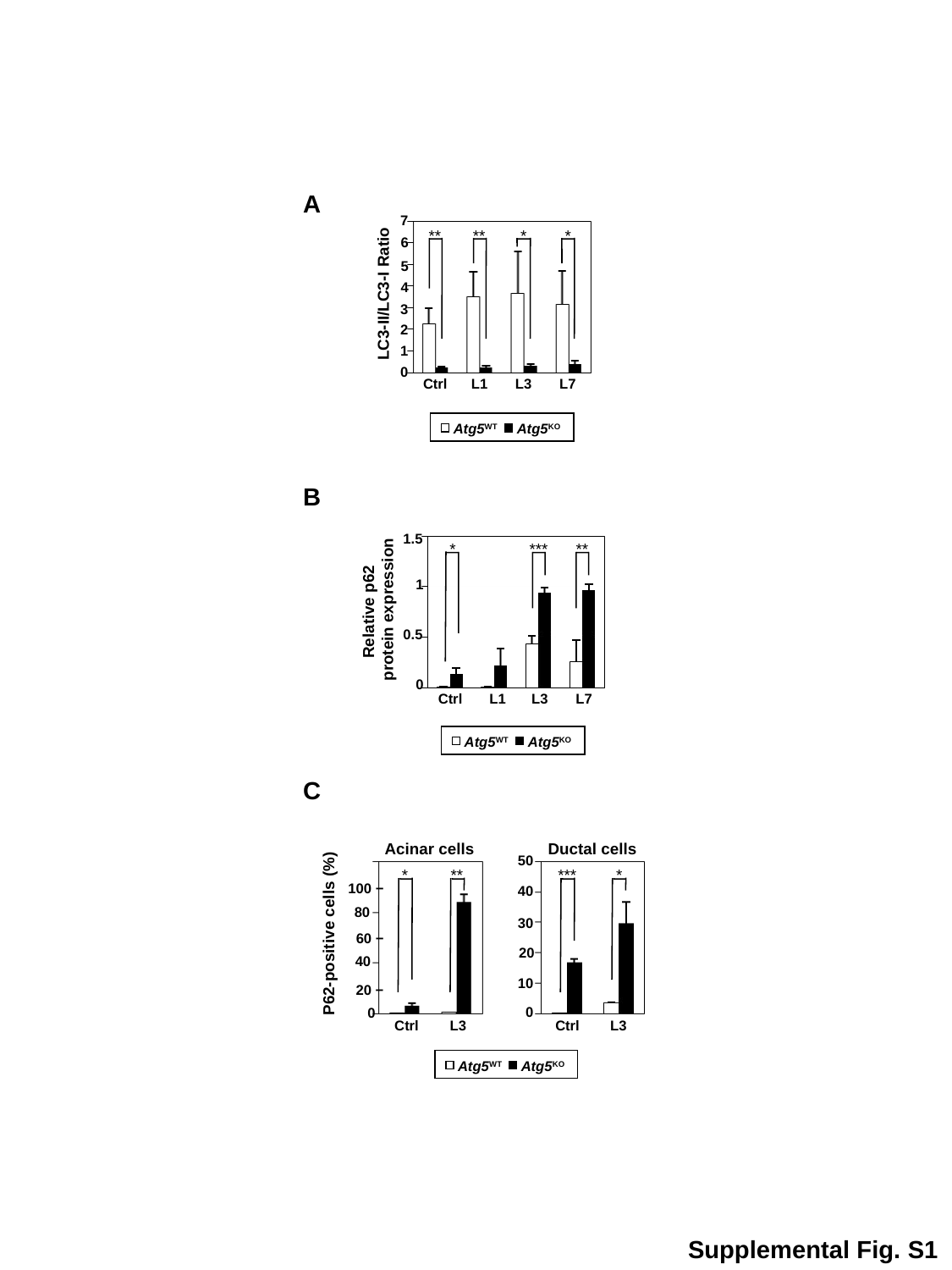

A
7
**
**
*
*
6
5
4
LC3-II/LC3-I Ratio
3
2
1
0
Ctrl
L1
L3
L7
Atg5WT
Atg5KO
B
1.5
*
***
**
1
Relative p62
protein expression
0.5
0
Ctrl
L1
L3
L7
Atg5WT
Atg5KO
C
Acinar cells
Ductal cells
50
*
**
***
*
40
80
30
P62-positive cells (%)
40
10
0
0
Ctrl
L3
Ctrl
L3
Atg5WT
Atg5KO
100 −
60 −
20
20 −
Supplemental Fig. S1
