# Supplementary material for: Dynamic involvement of ATG5 in cellular stress responses
Source: Cell Death Dis. 2014 Oct 23;5(10):e1478–. doi: 10.1038/cddis.2014.428 (PMC4649523; doi:10.1038/cddis.2014.428)
Supplement: Supplementary Figure S2 [file cddis2014428x3.ppt]

## Slide 1
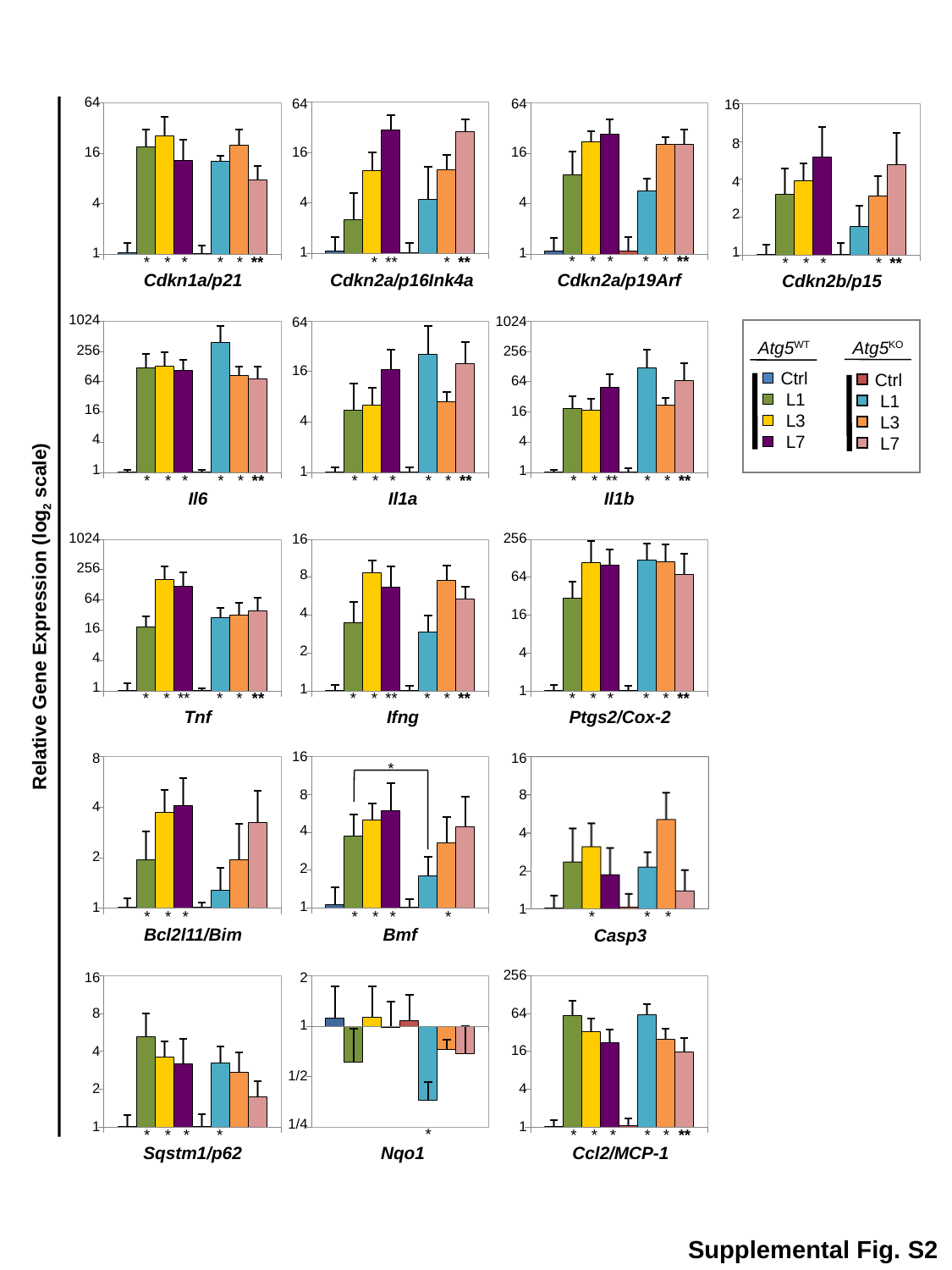

64
16
4
1
*
*
*
*
*
**
Cdkn1a/p21
64
16
4
1
*
*
*
*
*
**
64
16
4
1
16
8
4
2
1
*
*
*
*
**
Relative Gene Expression (log2 scale)
*
**
*
**
Cdkn2a/p16Ink4a
Cdkn2a/p19Arf
Cdkn2b/p15
1024
256
64
16
4
1
64
16
4
1
1024
256
64
16
4
1
Atg5KO
Ctrl
L1
L3
L7
Atg5WT
Ctrl
L1
L3
L7
*
*
*
*
*
**
Il6
*
*
*
*
*
**
Il1a
*
*
**
*
*
**
Il1b
1024
256
64
16
4
1
16
8
4
2
1
256
64
16
4
1
*
*
**
*
*
**
Tnf
*
*
**
*
*
**
Ifng
*
*
*
*
*
**
Ptgs2/Cox-2
16
8
4
2
1
8
4
2
1
16
8
4
2
1
*
*
*
Casp3
*
*
*
*
*
Bmf
*
*
*
Bcl2l11/Bim
16
8
4
2
1
2
1
1/2
1/4
256
64
16
4
1
*
Nqo1
*
*
*
*
Sqstm1/p62
*
*
*
*
*
**
Ccl2/MCP-1
Supplemental Fig. S2
