# Supplementary material for: Dynamic involvement of ATG5 in cellular stress responses
Source: Cell Death Dis. 2014 Oct 23;5(10):e1478–. doi: 10.1038/cddis.2014.428 (PMC4649523; doi:10.1038/cddis.2014.428)
Supplement: Supplementary Figure S3 [file cddis2014428x4.ppt]

## Slide 1
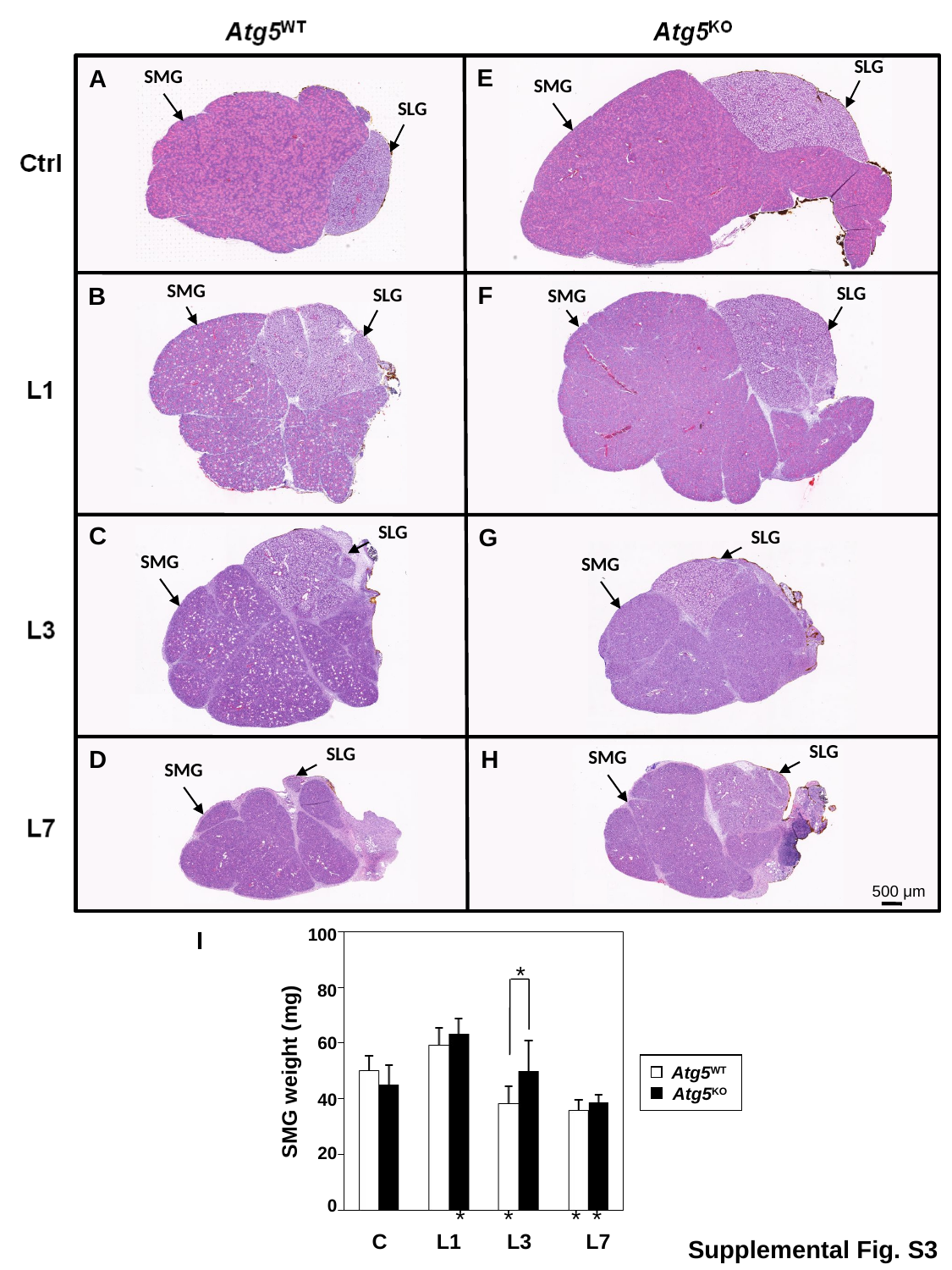

SLG
SMG
E
A
SMG
SLG
SMG
SLG
F
B
SLG
SMG
SLG
SMG
C
G
SLG
SMG
SLG
SMG
SLG
SMG
D
H
500 μm
100
80
60
40
20
0
I
*
SMG weight (mg)
Atg5WT
Atg5KO
*
*
*
*
C
L1
L3
L7
Supplemental Fig. S3
