# Supplementary material for: Dynamic involvement of ATG5 in cellular stress responses
Source: Cell Death Dis. 2014 Oct 23;5(10):e1478–. doi: 10.1038/cddis.2014.428 (PMC4649523; doi:10.1038/cddis.2014.428)
Supplement: Supplementary Figure S4 [file cddis2014428x5.ppt]

## Slide 1
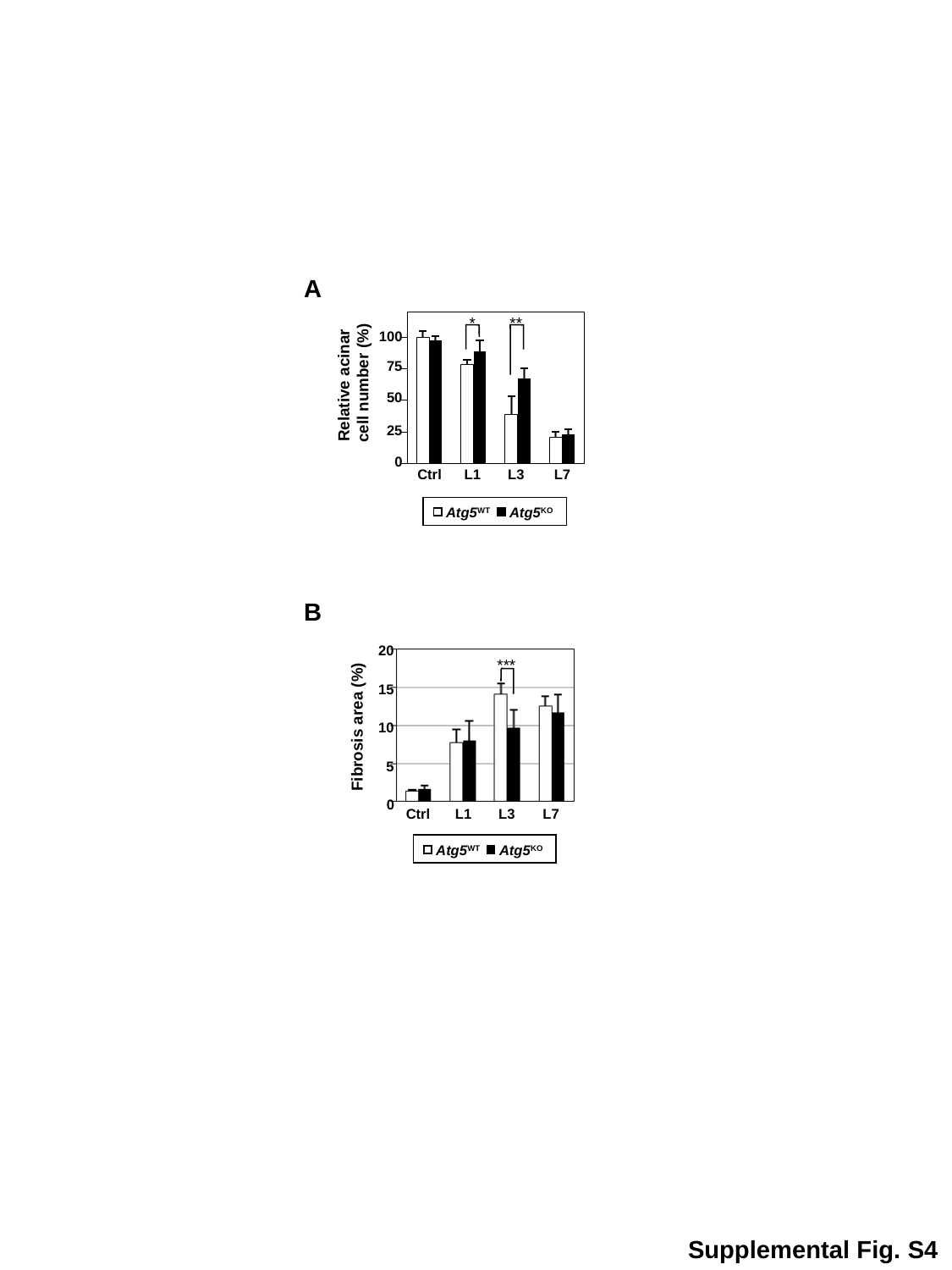

A
*
**
100
75
Relative acinar
cell number (%)
50
25
0
Ctrl
L1
L3
L7
Atg5WT
Atg5KO
B
20
***
15
Fibrosis area (%)
10
5
0
Ctrl
L1
L3
L7
Atg5WT
Atg5KO
Supplemental Fig. S4
