# Supplementary material for: Dynamic involvement of ATG5 in cellular stress responses
Source: Cell Death Dis. 2014 Oct 23;5(10):e1478–. doi: 10.1038/cddis.2014.428 (PMC4649523; doi:10.1038/cddis.2014.428)
Supplement: Supplementary Figure S5 [file cddis2014428x6.ppt]

## Slide 1
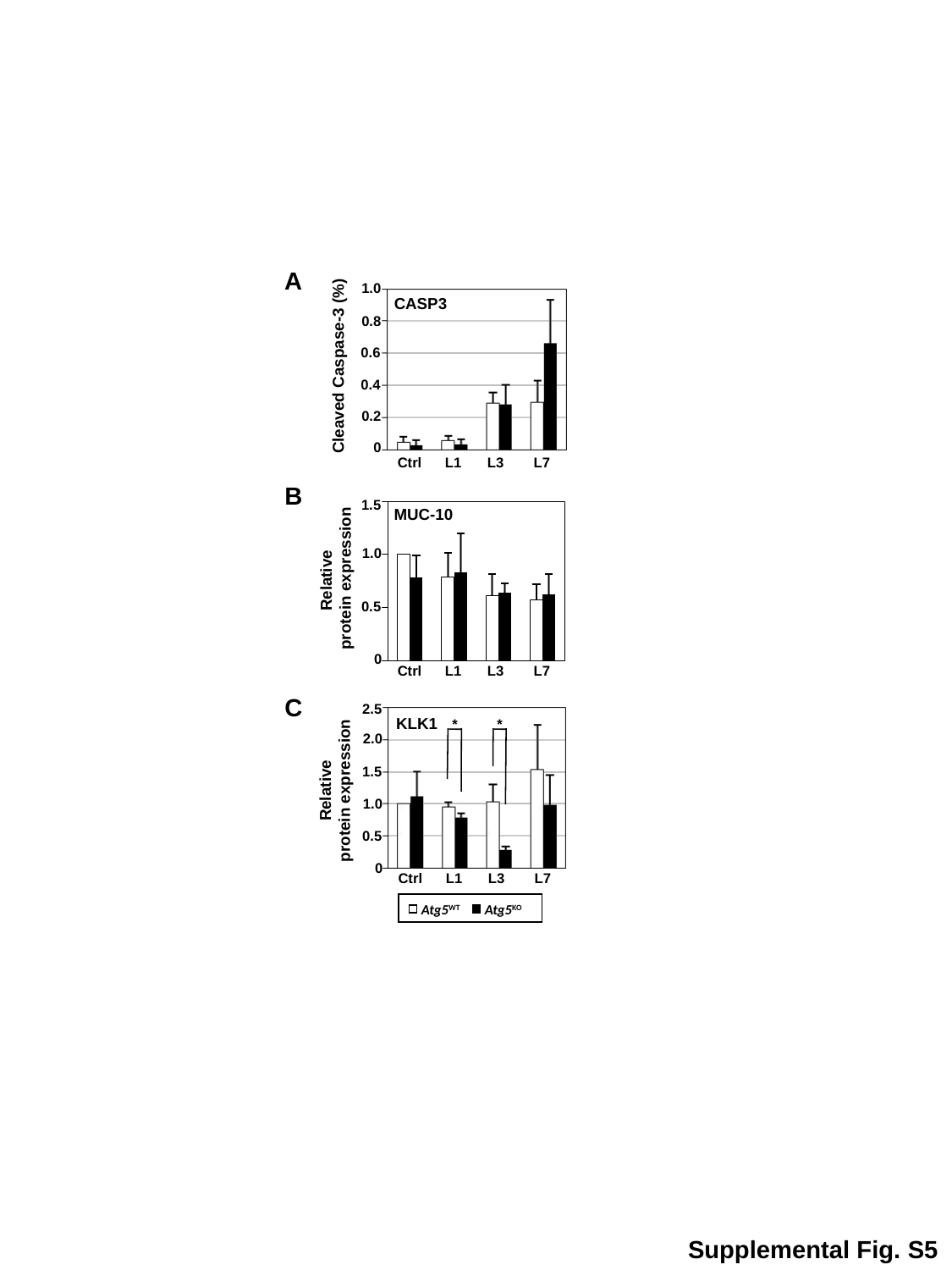

A
1.0
0.8
0.6
Cleaved Caspase-3 (%)
0.4
0.2
0
CASP3
Ctrl
L1
L3
L7
B
1.5
1.0
Relative
protein expression
0.5
0
MUC-10
Ctrl
L1
L3
L7
C
2.5
KLK1
*
*
2.0
1.5
Relative
protein expression
1.0
0.5
0
Ctrl
L1
L3
L7
Atg5WT
Atg5KO
Supplemental Fig. S5
