# Supplementary figures and images for: Dynamic involvement of ATG5 in cellular stress responses
Source: Cell Death Dis. 2014 Oct 23;5(10):e1478–. doi: 10.1038/cddis.2014.428 (PMC4649523; doi:10.1038/cddis.2014.428)

## Slide 1
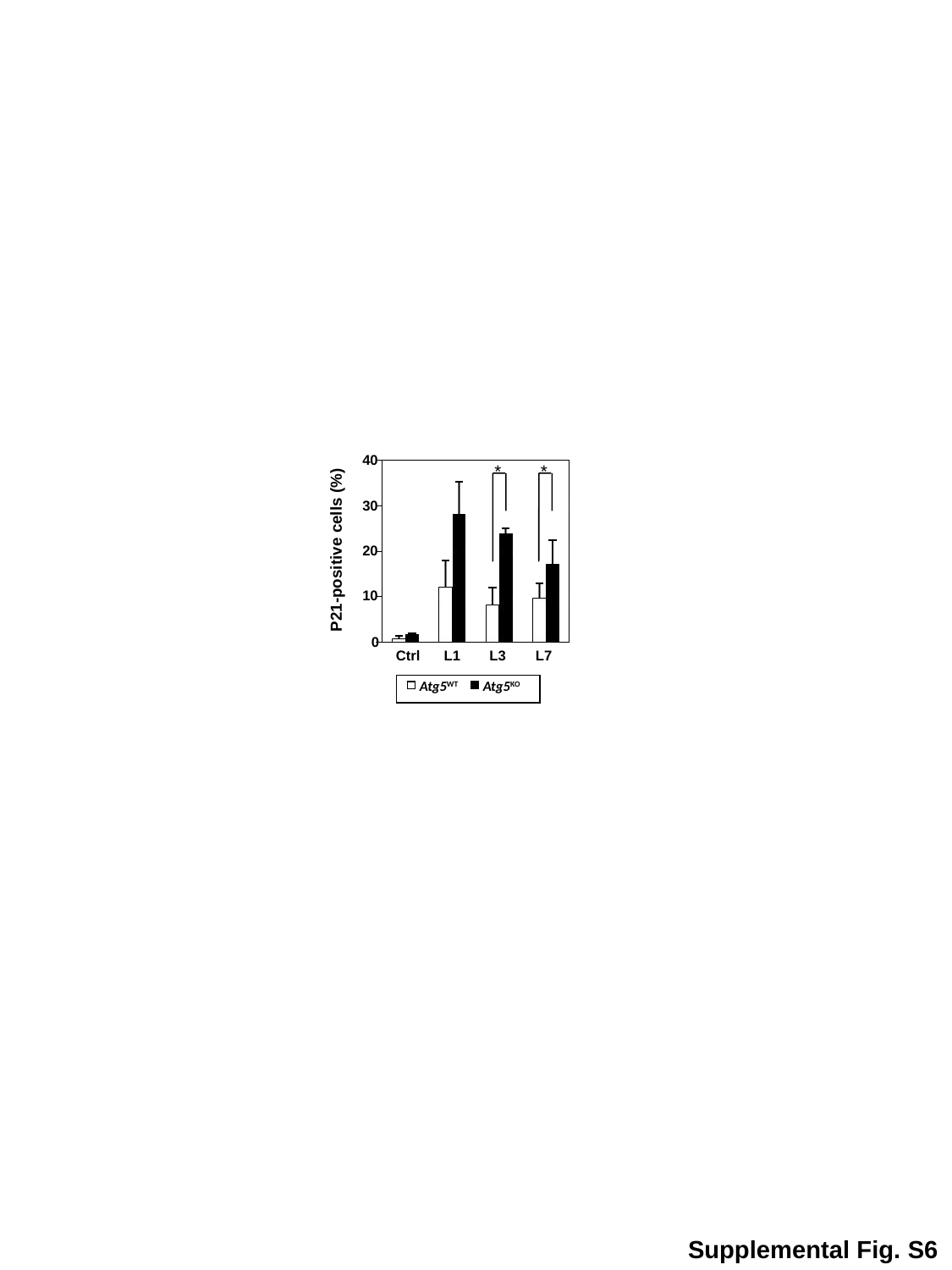

40
*
*
30
P21-positive cells (%)
20
10
0
Ctrl
L1
L3
L7
Atg5WT
Atg5KO
Supplemental Fig. S6

Supplement: Supplementary Figure S6 [file cddis2014428x7.ppt]

## Slide 1
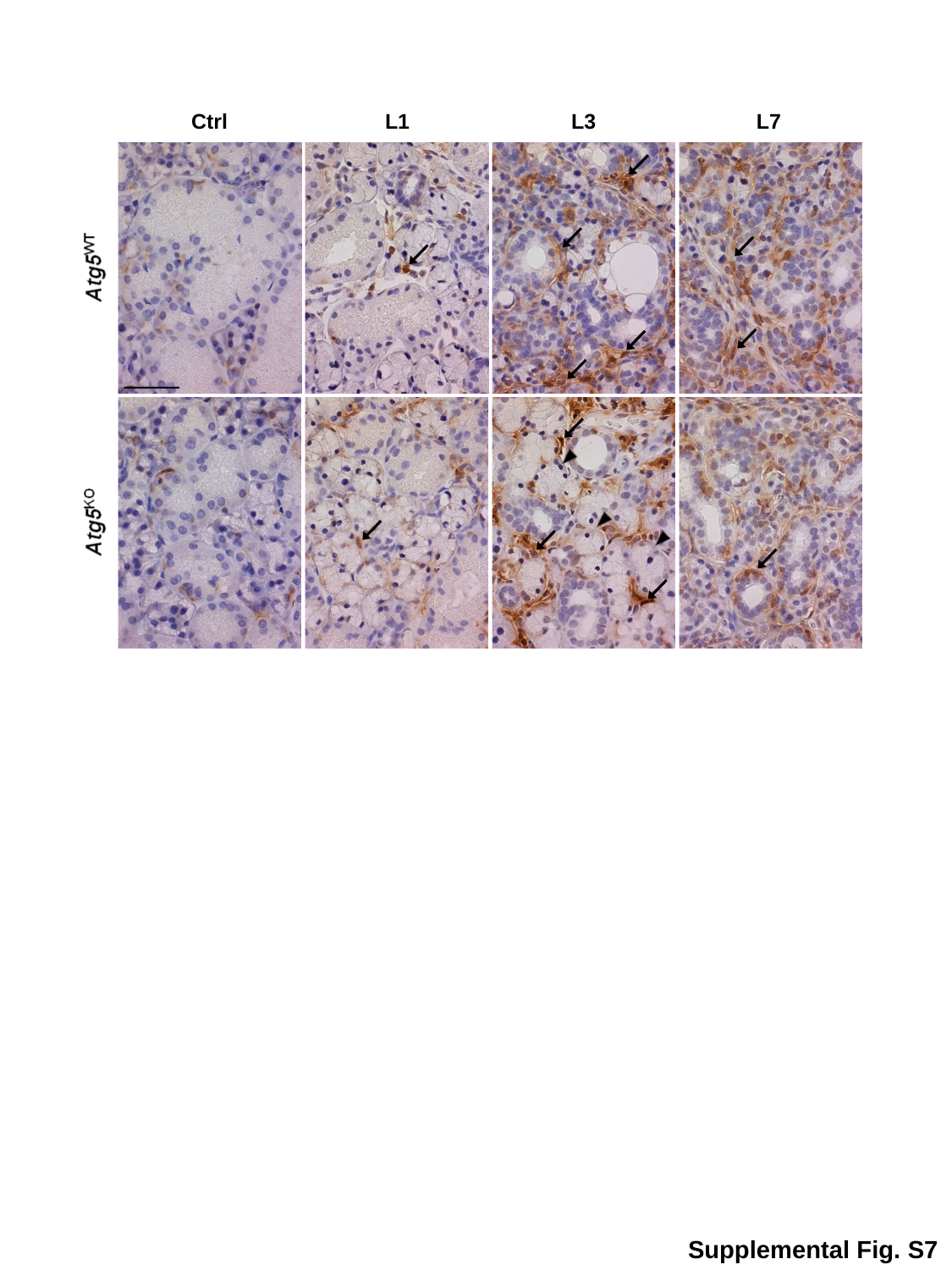

Ctrl
L1
L3
L7
Supplemental Fig. S7

Supplement: Supplementary Figure S7 [file cddis2014428x8.ppt]
